# Toward precision medicine in *SCN3A* variants-associated encephalopathies and epilepsy: optimizing genetic diagnosis and molecular subregional effects

**DOI:** 10.3389/fneur.2026.1772239

**Published:** 2026-02-05

**Authors:** Peng-Yu Wang, Jia-Xing Zhao, Wen-Hui Liu, Yong-Jun Chen, Hong-Wei Wang

**Affiliations:** 1 The Affiliated Nanhua Hospital, Department of Neurology, Hengyang Medical School, University of South China, Hengyang, China; 2 Department of Neurology, Institute of Neuroscience, Key Laboratory of Neurogenetics and Channelopathies of Guangdong Province and the Ministry of Education of China, The Second Affiliated Hospital, Guangzhou Medical University, Guangzhou, China; 3 Department of Neurology, The First Affiliated Hospital & Clinical Neuroscience Institute of Jinan University, Guangzhou, China; 4 The First Affiliated Hospital, Department of Neurology, Hengyang Medical School, University of South China, Hengyang, China

**Keywords:** algorithms, DEE, developmental and/or epileptic encephalopathies, ion channel, molecular subregional effects, optimized diagnostics, SCN3A

## Abstract

**Background:**

Variants in *SCN3A* gene encoding the voltage-gated sodium channel Nav1. 3 have been associated with severe developmental and/or epileptic encephalopathies, characterized by early-onset, drug-resistant seizures, malformations of cortical development, and profound neurodevelopmental impairment. Rapid clinical interpretation of *SCN3A* missense variants remains challenging. This study aimed to explore potentially reliable indicators in reflecting the pathogenicity of *SCN3A* variants, thereby promoting genetic diagnosis.

**Methods:**

The disease-associated and benign/likely benign *SCN3A* missense variants were systematically collected via two independent epilepsy geneticists to curate high-confidence dataset. The molecular subregional effects were analyzed to explore possible genotype-phenotype correlation. The diagnostic performance of nineteen commonly used algorithms was systematically evaluated using ROC analysis and confusion matrices metrics such as accuracy, sensitivity, specificity, and Matthews correlation coefficient (MCC).

**Results:**

A total of 20 pathogenic, affecting 37 patients, and 45 benign/likely benign *SCN3A* variants were included. Pathogenic *SCN3A* variants were statistically more located in transmembrane regions than in other regions, suggesting possible subregional effects. Deep-learning-based tools incorporating structural data, AlphaMissense, demonstrated superior balanced accuracy (>90%) and robust discrimination (AUC > 0.96). Meta-predictors, such as BayesDel_addAF and ClinPred, also showed high sensitivity but lower specificity at default thresholds. Notably, applying gene-specific optimal thresholds significantly improved performance across multiple tools.

**Conclusion:**

This study provides systematic benchmarks for algorithms in *SCN3A*-related DEEs. Integration of reliable algorithms with gene-specific thresholds into clinical variant interpretation pipelines could possibly refine the pathogenicity assessment of missense variants, subsequently informing timely risk stratification and personalized therapeutic strategies for affected patients.

## 1 Introduction

Developmental and epileptic encephalopathies (DEEs) constitute a group of severe neurodevelopmental disorders characterized by early-onset, drug-resistant seizures, malformations of cortical development, and profound neurodevelopmental impairment (1). These conditions typically manifest in infancy or early childhood, and in some cases, clinical or electrographic abnormalities are evident at birth or even prenatally. The phenotypic spectrum is broad, ranging from isolated developmental delay to profound neurological deficits accompanied by cortical malformations and movement disorders. Critically, the developmental dysfunction in DEEs is not solely attributable to seizure burden but also stems from the underlying pathogenic mechanism itself, which disrupts normal brain maturation. The profound impact of DEEs extends beyond the affected individuals, imposing substantial emotional, financial, and societal burdens on families and healthcare systems.

Recent advances in high-throughput genomic sequencing have accelerated the discovery of monogenic causes of DEEs. A growing number of genes have been associated with DEEs, including *KCNQ2* (2, 3), *SCN1A* (4), and *SCN2A* (5). Notably, many of the most frequently implicated genes encode voltage-gated ion channels, underscoring the critical dependence of early brain development on the precise regulation of neuronal excitability and network activity (6).

The *SCN3A* gene, located on chromosome 2q24, encodes sodium voltage-gated channel alpha subunit 3 (Nav1.3), which is mainly expressed in the brain and predominantly during the embryonic stage (7, 8). Nav1.3 proteins play crucial roles in the generation and propagation of action potentials or network maturation in the brain. In decades, pathogenic variants in *SCN3A* have been associated with an expanding spectrum of neurodevelopmental disorders, including familial focal epilepsy with variable foci-4 (FFEVF4 [MIM: 617935]) (7), epileptic encephalopathy-62 (DEE62 [MIM: 617938]) (8), and cortical malformations such as polymicrogyria and schizencephaly (9). Affected infants typically present with drug-resistant seizures, global developmental delay, and movement abnormalities. In severe patients, structural brain anomalies are detectable by prenatal or postnatal MRI. Most patients presented with early symptoms that resemble other forms of epilepsy. Even with genetic tests, pathogenicity cannot be assessed rapidly, especially for missense variants. Clinicians urgently require rapid and reliable methods for distinguishing pathogenic *SCN3A* variants from benign ones to support early diagnosis, risk stratification, and personalized therapy.

Among the various types of *SCN3A* variants, missense variants are difficult to interpret because the functional phenotypes cannot be inferred from gene dosage. To assist variant classification, numerous algorithms, such as SIFT (10, 11), PolyPhen-2 (12), MutationTaster (13), CADD (14), and REVEL (15), combine evolutionary conservation, physicochemical properties, structural modeling, and machine learning to estimate the likelihood that a given substitution is deleterious. Although these tools are embedded in most clinical sequencing pipelines, these algorithms are trained on genome-wide data and used by default thresholds that may not be optimal for *SCN3A* variants. Their predictive performance varies widely across genes and disease contexts (16, 17). Previous studies on *SCN1A* and *SCN2A* have demonstrated that prediction accuracy can be enhanced by integrating clinical phenotypes or protein structural constraints (18, 19). However, no systematic benchmark currently exists for *SCN3A*, which limits the optimizing genetic diagnosis of *SCN3A*-related disease.

In this study, we collected a curated set of pathogenic and benign *SCN3A* missense variants. The subregional effects of *SCN3A* were analyzed to screen the association between pathogenicity and the structure of the Nav1.3. Subsequently, A total of nineteen algorithms was analyzed including accuracy, sensitivity, specificity, and area under the receiver-operating characteristic curve (AUC). By providing vertical domain performance metrics and threshold recommendations, we aim to supply a systematic benchmark and framework that improves the clinical interpretation of *SCN3A* variants and patient care.

## 2 Materials and methods

### 2.1 Collection of patients and variants

To systematically assess the performance of algorithms in predicting the pathogenicity of *SCN3A* missense variants, we first initially classified variants into two categories: pathogenic and benign variants. Pathogenic variants were curated from the PubMed and HGMD database using the query: (*SCN3A* OR *EIEE62* OR *FFEVF4* OR *NAC3* OR Nav1.3) AND (variant OR mutation). Only variants supported by detailed clinical evidence and explainable origins were included (Supplementary Table S1). The benign variants were from the Genome Aggregation Database (gnomAD), which is denoted by the ClinVar database. Previously reported *SCN3A* variants and related phenotypes were systematically reviewed up to June 2025. All included variants met the criteria of the ACMG guidelines and were reviewed by a panel of neurogenetics experts and neurologists. These variants from real-world patients were deduplicated for further analysis.

### 2.2 Sub-molecular effects analysis

To investigate the sub-molecular determinants of pathogenicity, we used structural domain annotations from previous literature and mapped all curated missense variants onto the regions, including four domains with six transmembrane segments, the loops regions, and linker regions. The distribution of variants across these domains was then stratified by clinical interpretation. To assess whether specific domains/regions were enriched for pathogenic variants, we performed Fisher's exact test comparing the observed frequency of pathogenic variants within each domain against the population missense variants from gnomAD.

### 2.3 Algorithms prediction

Nineteen widely used and well-validated algorithms (Supplementary Table S1) were chosen for their proven effectiveness in assessing the functional consequences of missense variants. These tools included: AlphaMissense (20), BayesDel_addAF (21), BayesDel_noAF (21), ClinPred (22), ESM1b, Fathmm-XF_coding (23), LIST-S2 (24), M-CAP (25), MetaLR, MetaRNN, MetaSVM, MutationAssessor (26), MutationTaster (13, 27), Polyphen2_HDIV (28), Polyphen2_HVAR (28), PROVEAN (29), PrimateAI (30), SIFT (10), and SIFT4G (11). Scores were retrieved via precomputed database.

### 2.4 Performance evaluation by confusion matrices

To assess algorithms at its recommended thresholds, we built confusion matrices using a deduplicated dataset for the nineteen algorithms that provide classified variables. For binary outputs, “D” was defined as predicted pathogenic and “T” as predicted benign. Multi-class outputs were defined as follows: PolyPhen-2 (HDIV and HVAR)—“D” and “P” as pathogenic, “B” as benign; MutationTaster—“A” or “D” as pathogenic, “N” or “P” as benign; MutationAssessor—“H” or “M” as pathogenic, “N” or “L” as benign; AlphaMissense—“P” as pathogenic, “B” or “A” as benign; Fathmm-XF_coding—“D” as pathogenic, “N” as benign; PROVEA—“D” as pathogenic, “N” as benign; *SCN3A* missense variants were then categorized into true positives (TP), true negatives (TN), false positives (FP), and false negatives (FN).

Based on these basic metrics, we calculated additional performance indicators, including accuracy, sensitivity, specificity, positive predictive value (PPV), and negative predictive value (NPV). Furthermore, we evaluated the *F*-score, a harmonic mean of precision and recall, and the Matthews correlation coefficient (MCC), a robust measure of binary classification quality. The *F* score was computed as $\frac{2PR}{P\;+\;R}$, where precision (*P*) = $\frac{TP}{TP\;+\;FP}$ and recall (*R*)= $\frac{TP}{TP\;+\;FN}$. The MCC was determined using the formula: $\frac{TP\;\times \;TN\;-\;FP\;\times \;FN}{\sqrt{\left(TP\;+\;FP\right)\left(TP\;+\;FN\right)\left(TN\;+\;FP\right)\left(TN\;+\;FN\right)}}$. This coefficient ranges from −1 to 1, with −1 representing a completely incorrect prediction, 0 indicating random performance, and 1 denoting a perfectly accurate classifier.

### 2.5 Statistical analysis and ROC curve

Score distributions were first compared between pathogenic and benign variants. Normality in each group was checked with the Shapiro–Wilk test; data that were normal with equal variances were analyzed with Student's *t*-test, while data with unequal variances were analyzed with Welch's *t*-test, and non-normal data with the Wilcoxon rank-sum test. A *p*-value < 0.05 was considered statistically significant for all comparisons.

To compare algorithms of thresholds independently, algorithms with significant differences were included in the ROC analysis. The area under the curve was calculated with the DeLong method and is reported with its 95% confidence interval and the *p*-value vs. 0.5. The threshold that maximized Youden's index defined the optimal operating point; at this point, we extracted the full confusion matrix and derived accuracy, sensitivity, specificity, positive predictive value, negative predictive value, Matthews correlation coefficient, and *F*1-score. Higher AUC values indicated better discriminatory power for distinguishing pathogenic from benign *SCN3A* variants. All statistical analyses and visualizations were carried out in R (version 4.5.2) using the packages dplyr, purrr, pROC, janitor, and ggplot2.

## 3 Results

### 3.1 Molecular subregional effects analysis of *SCN3A* variants

**Figure 1:**
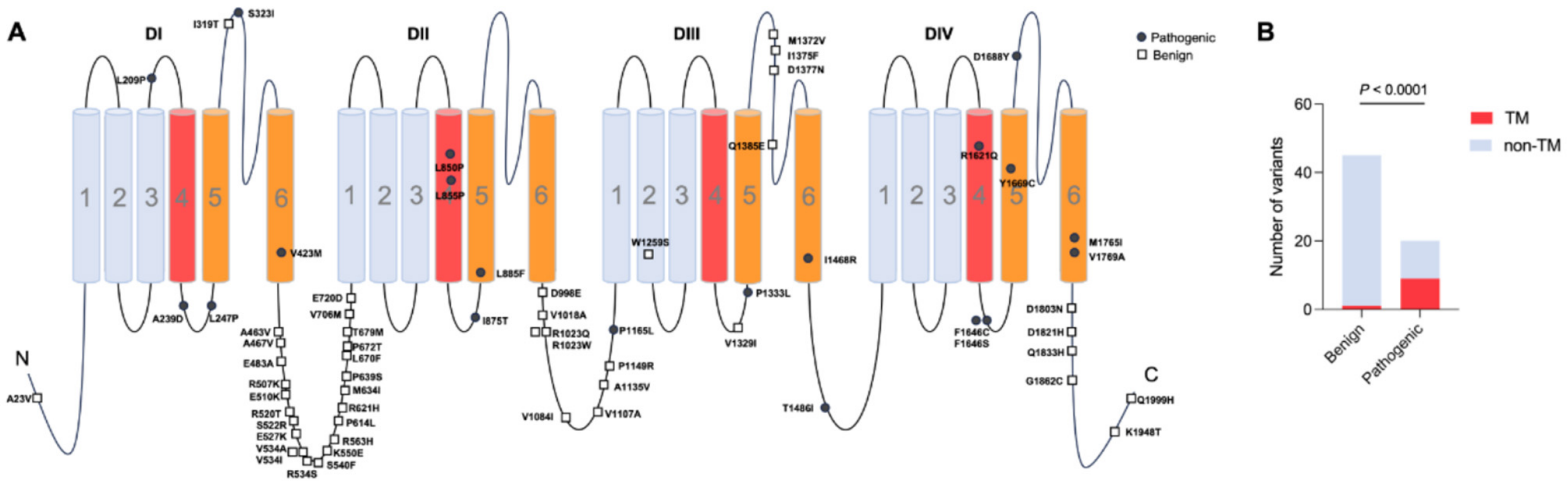
Molecular subregional effects analysis of *SCN3A* variants. **(A)** The locations of *SCN3A* variants were associated with the pathogenicity of variants. **(B)** Patients with *SCN3A* variants in the transmembrane regions showed a significantly higher prevalence of pathogenic variants compared to those with variants in other regions.

We systemically reviewed the location of previously reported *SCN3A* variants and their pathogenicity and associated phenotypes (including whether they presented with seizures or polymicrogyria) (8, 9, 31–41). To date, a total of 20 pathogenic variants from 37 patients and 45 benign/likely benign *SCN3A* variants have been identified (Table 1), which were associated with varied pathogenicity. To analyze the molecular sub-regional effects, the variants were classified as pathogenic and benign, which were consistent with the above test set. It is shown that pathogenic *SCN3A* variants were statistically more located in transmembrane regions than in other regions (Figures 1A, B). Considering the phenotypic heterogeneity, we tested the association between variant location and clinical presentation. No significant correlations were observed between positional features and phenotype, including seizures or polymicrogyria (Supplementary Figure S1). Different regions or domains showed varied toleration to missense variants, which served as a useful indicator for predicting missense pathogenicity. These results suggest that structural annotations helped identify pathogenicity but provide limited value for classifying clinical subtypes. This finding warrants the need to integrate clinical covariates when annotating *SCN3A* variants to develop gene-specific, vertical models with greater clinical utility.

**Table 1:** Characteristics of previously reported cases with putative disease-causing *SCN3A* variants.

**Variant**	**cDNA**	**Sex**	**EEG**	**Brain MRI**	**Development and other**	**Diagnosis**	**inherited**	**AF-v4**	**AF-v2**	**Source**
p.Leu209Pro	c.626T>C	M	–	Abnormal	ID; dystonia	NDD without EP	*De novo*	–	–	30542205
p.Ala239Asp	c.716C>A	M	–	–	GDD	DEE	*De novo*	–	–	34055682
p.Leu247Pro	c.740T>C	F	Multifocal	Normal	GDD; dystonia	DEE	*De novo*	–	–	28235671
p.Ser323Ile	c.968G>T	M	–	–	GDD	DEE	*De novo*	–	–	34145886
p.Val423Met	c.1267G>A	M	–	–	DD; dystonia	NDD^*^	*De novo*	–	–	34490615
p.Leu850Pro	c.2549T>C	M	–	PMG	GDD; dystonia	NDD without EP	*De novo*	–	–	30146301
p.Leu855Pro	c.2564T>C	F	–	–	GDD; dystonia; spastic tetraplegia	NDD without EP	*De novo*	–	–	32515017
p.Ile875Thr	c.2624T>C	M	–	PMG	DD	DEE	*De novo*	–	–	29466837
p.Ile875Thr	c.2624T>C	F	–	PMG	DD	DEE	*De novo*	–	–	29466837
p.Ile875Thr	c.2624T>C	M	–	PMG, microcephaly	NDD; hypotonia; spastic tetraplegia	DEE	*De novo*	–	–	30146301
p.Ile875Thr	c.2624T>C	F	–	PMG, microcephaly	GDD; hypotonia; spastic tetraplegia	DEE	*De novo*	–	–	30146301
p.Ile875Thr	c.2624T>C	M	Multifocal	PMG	ID; dystonia	DEE	*De novo*	–	–	32515017
p.Ile875Thr	c.2624T>C	F	Multifocal	PMG	DD; hypotonia; spastic tetraplegia	DEE	*De novo*	–	–	32515017
p.Ile875Thr	c.2624T>C	F	Multifocal	PMG	Hypotonia; DD	DEE	*De novo*	–	–	32515017
p.Ile875Thr	c.2624T>C	M	–	PMG	ID; hypotonia; spastic	DEE	*De novo*	–	–	32515017
p.Ile875Thr	c.2624T>C	M	Multifocal	PMG	DD; hypotonia	DEE	*De novo*	–	–	32515017
p.Ile875Thr	c.2624T>C	M	Multifocal	PMG	GDD	DEE	*De novo*	–	–	29740860
p.Ile875Thr	c.2624T>C	M	Hypsarrhythmia, multifocal	PMG	GDD	DEE	*De novo*	–	–	29740860
p.Leu885Phe	c.2653C>T	F	Burst suppression, hypsarrhythmia	Frontal pachygyria; hypoplastic corpus callosum	DD; hypotonia; spastic tetraplegia	DEE	*De novo*	–	–	32515017
p.Pro1165Leu	c.3494C>T	F	Focal discharge	–	Normal	FE	Inherited from affected mother	0.000008058	0	34992632
p.Pro1333Leu	c.3998C>T	M	Hypsarrhythmia, multifocal	Normal	DD	DEE	*De novo*	–	–	29466837
p.Pro1333Leu	c.3998C>T	M	Hypsarrhythmia, multifocal	Hypoplastic corpus callosum	DD; hypotonia	DEE	*De novo*	–	–	32515017
p.Pro1333Leu	c.3998C>T	NA	–	–	DD	DEE	*De novo*	–	–	27848944
p.Ile1468Arg	c.4403T>G	F	Hypsarrhythmia, multifocal	Normal	DD; hypotonia	DEE	*De novo*	–	–	32515017
p.Thr1486Ile	c.4457C>T	M	Generalized discharges	PMG	Pseudobulbar palsy; ID	DEE	*De novo*	–	–	32515017
p.Arg1621Gln	c.4862G>A	M	Normal	PMG	ID	DEE	*De novo*	–	–	32515017
p.Arg1621Gln	c.4862G>A	M	–	PMG	GDD; aggressive behavior; ADHD	DEE	*De novo*	–	–	31618753
p.Phe1646Ser	c.4937T>C	F	Multifocal	PMG	Dysarthria; facial paresis; brisk reflexes; ID	DEE	*De novo*	–	–	32515017
p.Phe1646Cys	c.4937T>G	M	–	PMG	Pseudobulbar palsy; right hemiparesis; DD	NDD without EP	Inherited from affected mother	–	–	32515017
p.Phe1646Cys	c.4937T>G	F	–	PMG	Pseudobulbar palsy; brisk reflexes; ID	DEE	Inherited from affected mother	–	–	32515017
p.Tyr1669Cys	c.5006A>G	M	–	–	DD; ASD	NDD without EP	*De novo*	0.000001239	–	32515017
p.Asp1688Tyr	c.5062G>T	F	Multifocal	Normal	Normal	FE	Inherited from affected father	0.0000006195	0	33895391
p.Met1765Ile	c.5295G>A	M	–	PMG	hypotonia; DD.	DEE	*De novo*	–	–	32515017
p.Met1765Ile	c.5295G>A	M	Multifocal	PMG, hypoplastic corpus callosum	GDD; dyskinetic movements; cortical blindness; spastic tetraplegia	DEE	*De novo*	–	–	31677917
p.Val1769Ala	c.5306T>C	F	Multifocal	–	DD	DEE	*De novo*	–	–	29466837
p.Val1769Ala	c.5306T>C	F	Multifocal	–	DD	DEE	*De novo*	–	–	32515017
p.Val1769Ala	c.5306T>C	F	Hypsarrhythmia or generalized discharges	Normal	DD; hypotonia; ASD	DEE	*De novo*	–	–	32515017

P^*^, undetermined diagnosis of EP; – or NA, not available; ADHD, attention deficit hyperactivity disorder; AF-v2, allele frequencies from gnomAD v2; AF-v4, allele frequencies from gnomAD v4; ASD, autism spectrum disorder; DD, developmental delay; EP, epilepsy; F, female; FE, focal epilepsy; ID, intellectual disability; M, male; NDD, neurodevelopmental disorders; PMG, polymicrogyria; Source, PubMed ID.

### 3.2 Performance evaluation of algorithms tools

The predictive performance of nineteen widely used algorithms were assessed at recommended thresholds, to establish the systematic benchmark and evaluation framework for *SCN3A* variants. Variants were carefully curated: pathogenic variants were obtained from a trusted source and supported by a confirmed clinical diagnosis reviewed by our panel, while benign variants were retrieved from the gnomAD database, confirming their non-deleterious nature.

**Figure 2:**
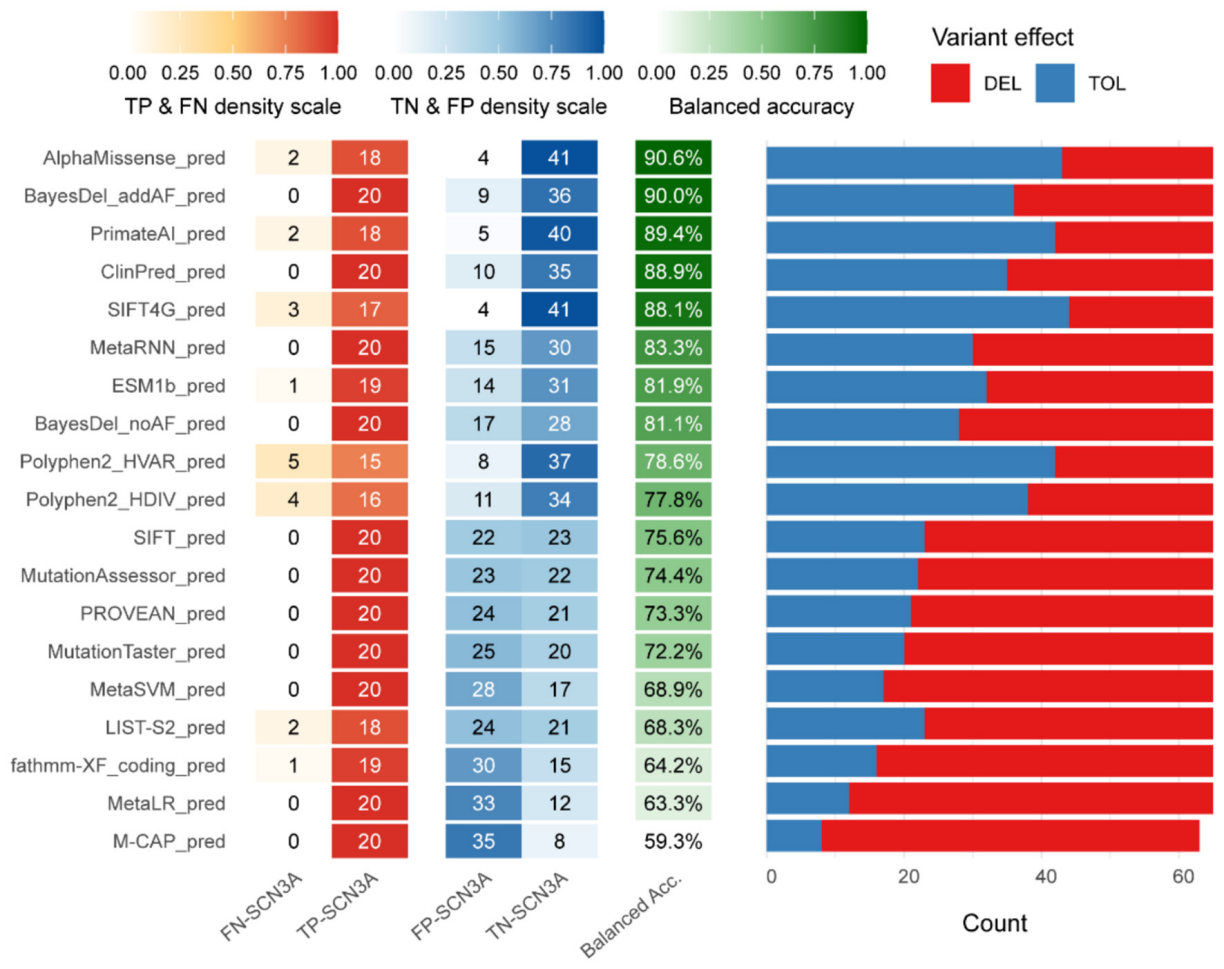
Algorithms performance evaluation. The left heatmap showed the classification outcomes: true positives (TP) and false negatives (FN) among pathogenic variants, and true negatives (TN) and false positives (FP) among benign variants. The central column showed balanced accuracy. The balanced accuracy of each algorithm served as the primary metric to rank the algorithms. The distribution of predictions made by each algorithm at its recommended threshold, showing the proportion of variants classified as deleterious (DEL, red) vs. tolerated (TOL, blue).

The nineteen algorithms that provide classified output showed differences in performance (Figure 2 and Supplementary Table S2). AlphaMissense and PrimateAI, two deep-learning models trained by structure information, achieved the most robust result. AlphaMissense achieved the highest balanced accuracy of 90.6% and a superior MCC of 0.7911, followed closely by PrimateAI with a balanced accuracy of 89.4% and a MCC of 0.7615. Both maintained a balance between sensitivity (>0.90) and specificity (>0.88), suggesting that structural information effectively minimizes false positives. Two meta-predictors, BayesDel_addAF (90.0%) and ClinPred (88.9%), were also showed high balanced accuracy, yet their performance profile differed; While both achieved a perfect sensitivity of 1.0, their lower specificities (0.80 and 0.7778) reflect a strategic bias toward “over-calling” pathogenicity to ensure no deleterious variants are missed. This trend continued in MetaRNN and BayesDel_noAF, where balanced accuracy remained above 81% but MCC values were below 0.62, indicating a loss in overall predictive reliability. Among tools relying primarily on sequence conservation and traditional machine learning methods, SIFT4G performed with a balanced accuracy of 88.06%. Polyphen2_HVAR and Polyphen2_HDIV exhibited intermediate results, with balanced accuracy of 78.6% and 77.8%, respectively, both showing sensitivities around 0.75–0.8 but higher false negatives. PROVEAN and MutationTaster showed balanced accuracy decline toward 72% due to specificities falling below 0.47. The lowest performance was observed in M-CAP, which yielded a balanced accuracy of only 59.3%, with a specificity of 0.186, offering limited utility for filtering benign variants.

### 3.3 Difference analysis and ROC curves for the algorithms tools

**Figure 3:**
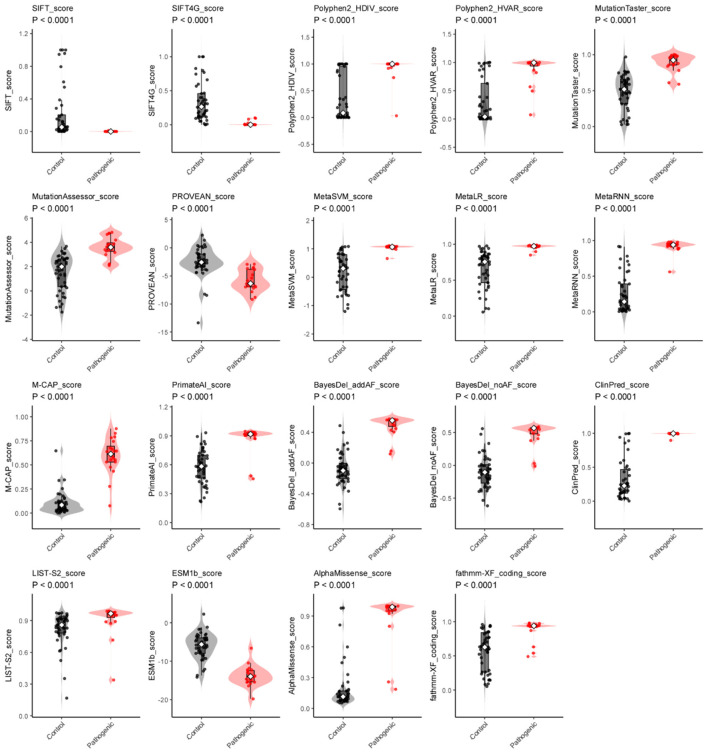
Difference analysis of algorithms prediction scores for *SCN3A* variants. For each algorithm (including AlphaMissense, BayesDel_addAF, BayesDel_noAF, ClinPred, ESM1b, Fathmm-XF_coding, LIST-S2, M-CAP, MetaLR, MetaRNN, MetaSVM, MutationAssessor, MutationTaster, Polyphen2_HDIV, Polyphen2_HVAR, PROVEAN, PrimateAI, SIFT, and SIFT4), scores are shown for benign (gray) and pathogenic (red) variants. Violin plots show how the scores are distributed; The difference between groups was tested with a Wilcoxon rank-sum test; all were highly significant (*p* < 0.0001).

Difference analysis of scores among algorithms (including derived algorithms) showed clear separation between pathogenic and benign (control) variants (Figure 3). All comparisons were significant (*p* < 0.0001).

**Figure 4:**
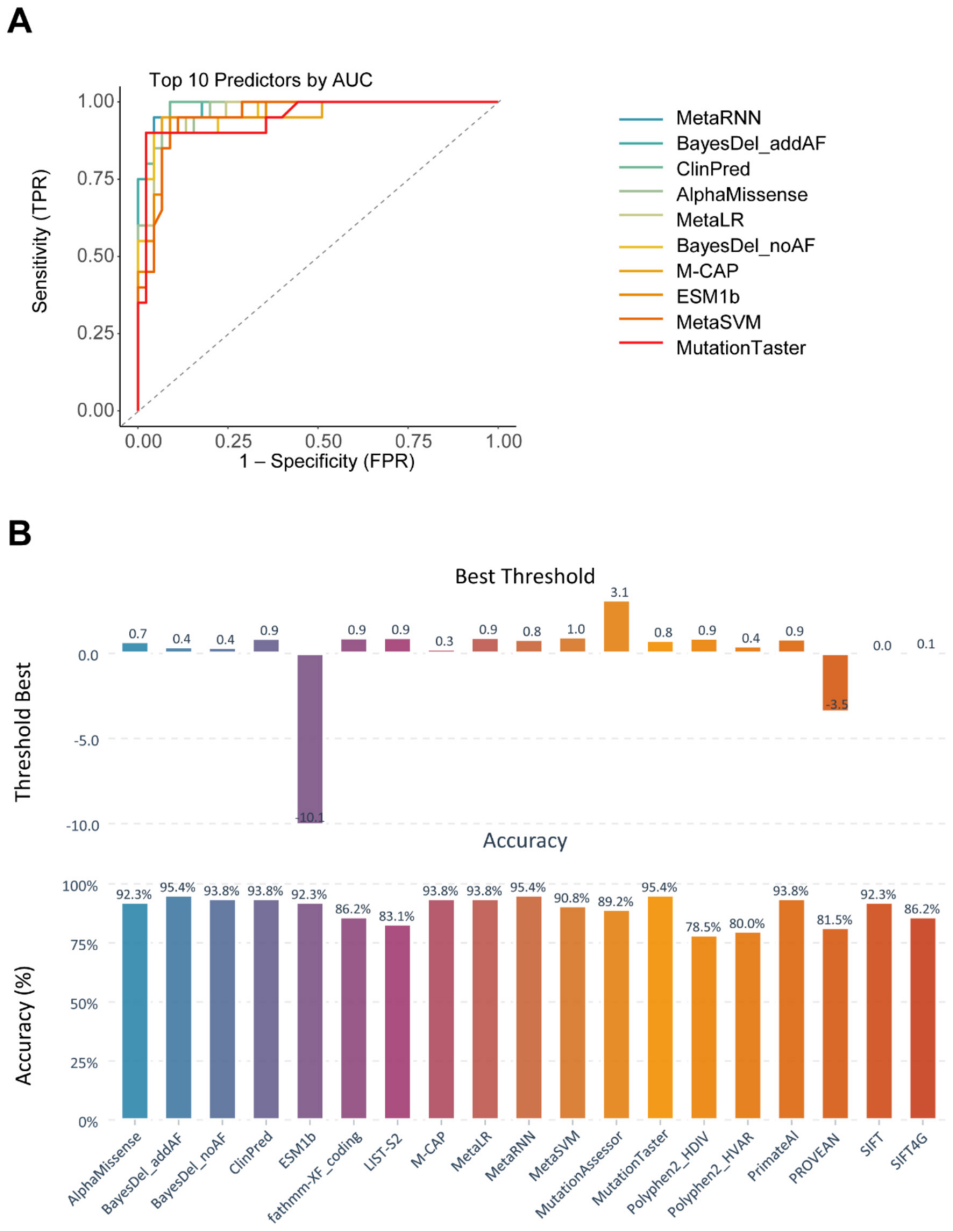
Receiver operating characteristic curve (ROC) performance of top ten algorithms and optimal thresholds of algorithms for *SCN3A* variants. **(A)** Each algorithm is represented by different colors, and the dashed line indicates random distribution. The area under each curve (AUC), along with its 95% confidence interval (CI), is shown, higher AUC values reflect better discrimination between pathogenic and benign variants. Full ROC curves for all tools are provided in Figure 5. **(B)** Bar plot showed the data-driven best threshold for each tool (top) and the accuracy at that threshold (bottom). Best thresholds were defined by maximizing Youden's J. Supplementary Table S3 listed sensitivity, specificity, balanced accuracy, *F*1-score, and MCC, and optimal thresholds.

**Figure 5:**
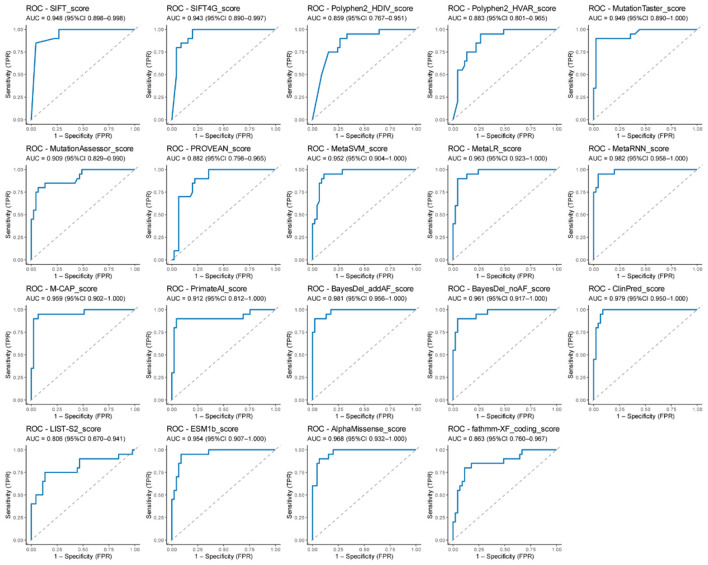
All ROC curves across nineteen algorithms. ROC curves were presented with AUC and 95% CI, including AlphaMissense, BayesDel_addAF, BayesDel_noAF, ClinPred, ESM1b, Fathmm-XF_coding, LIST-S2, M-CAP, MetaLR, MetaRNN, MetaSVM, MutationAssessor, MutationTaster, Polyphen2_HDIV, Polyphen2_HVAR, PROVEAN, PrimateAI, SIFT, and SIFT4.

ROC analysis further showed performance differences among algorithms (Figure 4A and Supplementary Table S3). AUCs ranged from 0.81 to 0.98 with a median of 0.95. The top-performance algorithms by ROC analysis were MetaRNN (AUC [0.982], 95% CI [0.958–1]), BayesDel_addAF (AUC [0.981], 95% CI [0.956–1]), ClinPred (AUC [0.979], 95% CI [0.95–1]), and AlphaMissense (AUC [0.968], 95% CI [0.932–1]; Figure 5) Best thresholds were calculated by Youden's J statistic; Most algorithms achieved performance improvements at the optimal threshold (Figure 4B and Supplementary Table S3). Compared with published/recommended cutoffs, majority of algorithms achieved better performance than the others at the recommended threshold. For example, AlphaMissense showed best thresholds differed at the threshold of 0.699 and improved accuracy with sensitivity of 0.9, specificity of 0.933, MCC of 0.822, and *F*-score of 0.878. BayesDel_addAF showed best thresholds differed at the threshold of 0.401 and improved accuracy with sensitivity of 0.9, specificity of 0.978, MCC of 0.891, and F-score of 0.923. Notably, M-CAP, MetaLR, and fathmm-XF_coding largely improved accuracy after adjusting the threshold.

## 4 Discussion

In this study, we collected a curated set of pathogenic and benign *SCN3A* missense variants. Pathogenic *SCN3A* variants were statistically more located in transmembrane regions than in other regions, suggesting possible subregional effects. Systematic benchmarks at their recommended thresholds were provided by building confusion matrices and comparing the balanced accuracy, sensitivity, specificity, MCC, and *F*1-score. AlphaMissense achieved the highest performance. Statistical analysis showed significant differences between pathogenic and benign variants across all algorithms, and ROC curves were then used to assess threshold-independent predictive performance of algorithms.

Features considered include evolutionary conservation, characteristics of the protein structure, properties of the amino acids involved, underlying nucleotide-level attributes, and patterns of variation observed in clinical and population databases. In benchmarks at recommended thresholds, AlphaMissense and PrimateAI, which include structural information, achieved high balanced accuracy. SIFT4G, driven almost exclusively by conservation scores, already reached a generally acceptable level, and BayesDel_addAF outperformed BayesDel_noAF, confirming the added value of allele-frequency data. These findings underscore that conservation and population frequency are core indicators of variant impact, while structural information can further raise predictive performance. This study warrants further attribution analysis for these tools, such as SHapley Additive exPlanations (SHAP), which quantifies the contribution of each feature group and thus informs rational feature selection and algorithm refinement.

With the recommended threshold, multiple prediction algorithms achieved high accuracy in distinguishing between pathogenic and benign *SCN3A* variants, including, AlphaMissense, BayesDel_addAF, PrimateAI, and ClinPred. However, the optimal tool may vary depending on the specific clinical or research. For scenarios in which sensitivity is paramount, such as initial screening or when the primary aim is to minimize false negatives, ClinPred is particularly suitable with a sensitivity of 1.00 at both thresholds, ensuring that nearly all pathogenic variants are identified. In contrast, when specificity is the primary consideration, for example, in confirmatory analyses where minimizing false positives is crucial, AlphaMissense demonstrated outstanding performance, with a specificity of 0.91 at recommend threshold. For routine clinical use, where balanced performance between sensitivity and specificity is most desirable, AlphaMissense, PrimateAI, BayesDel_addAF, and ClinPred provided high accuracy and balanced accuracy, making them strong candidates for general application. Notably, the performance metrics observed here represent a significant improvement over earlier benchmarks for sodium channels, which typically reported accuracies in the range of 0.50–0.80. This improvement reflects the development of machine learning algorithms. In addition, previous studies on *SCN1A, SCN2A*, and *SCN8A* have already demonstrated that gene-specific optimization enhanced the performance of traditional methods. Our findings on *SCN3A* corroborate this pattern, confirming that tailored calibration is as critical as algorithm selection for accurate variant interpretation. These findings suggest that algorithm selection should be tailored to the practical demands of different use cases in variant interpretation.

ROC analysis demonstrated that multiple algorithms, such as MetaRNN, BayesDel_addAF, ClinPred, and AlphaMissense, exhibited a favorable balance between predictive efficiency, sensitivity, and specificity. Notably, M-CAP, MetaLR, and fathmm-XF_coding demonstrated a remarkable improvement in prediction performance when gene-specific optimal thresholds were applied, differing from previous benchmarking results based on thresholds without gene stratification. These findings highlight a general improvement in predictive performance across most algorithms when gene-level thresholds are adopted. This study underscored the value of refining classification criteria at the gene level and emphasized the importance of establishing gene-specific vertical evaluation metrics.

Previous studies showed that the damaging effects of variants were associated with gene-specific sub-molecular effects (42–48). Variants may lead to heterogeneous phenotypes related to domain, residue burial or depth from the protein surface, proximity to functional sites, and biophysical changes of ion channels. In this study, missense variants within the transmembrane domain were more often pathogenic than those in the non-transmembrane domain, which indicated regional heterogeneity of missense intolerance within *SCN3A*. Domain or structural location may serve as a predictor of pathogenicity. A key advantage of a vertical, gene-specific framework is that such sub-molecular features or other functional features may be explicitly integrated into prediction models to improve performance.

Several aspects of this study warrant consideration. First, given the inherent rarity of *SCN3A*-related diseases, the dataset size is relatively constrained, which may influence the precision of performance estimates. Consequently, the current benchmarking results should be viewed as a foundational framework. Second, while gene-specific thresholds demonstrated improved performance, future validation in independent, large-scale cohorts would further strengthen their generalizability and mitigate potential overfitting risks. Finally, this study contrasted clearly pathogenic variants with population controls; extending this analysis to variants with intermediate phenotypes or lower penetrance in future studies may help in characterizing algorithmic performance across the phenotypic spectrum.

## Data Availability

The original contributions presented in the study are included in the article/Supplementary material, further inquiries can be directed to the corresponding author/s.

## Supplementary material

Supplementary Figure S1 Molecular subregional effects analysis of *SCN3A* variants. **(A)** Schematic representation of the Nav1.3 proteins structure highlighting the positions of variants associated with developmental and epileptic encephalopathy (DEE) and neurodevelopmental disorders (NDD) without epilepsy phenotype (EP). **(B)** Bar chart comparing the number of variants in S4-S6 segments vs. other segments for patients with NDD with EP and DEE. No significant was shown. **(C)** The location of variants associated with polymicrogyria (PMG), normal, and other phenotypes. **(D)** Bar chart comparing the number of variants in transmembrane (TM) segments vs. non-transmembrane (non-TM) segments for patients with PMG and normal individuals. No significant was shown.

Supplementary Table S1 Detailed description of algorithms.

Supplementary Table S2 Performance valuation of nineteen algorithms at the recommended threshold.

Supplementary Table S3 Performance valuation of nineteen algorithms at the best threshold.
