# Supplementary figures and images for: Toward precision medicine in SCN3A variants-associated encephalopathies and epilepsy: optimizing genetic diagnosis and molecular subregional effects
Source: Front Neurol. 2026 Feb 5;17:1772239. doi: 10.3389/fneur.2026.1772239 (PMC12916406; doi:10.3389/fneur.2026.1772239)

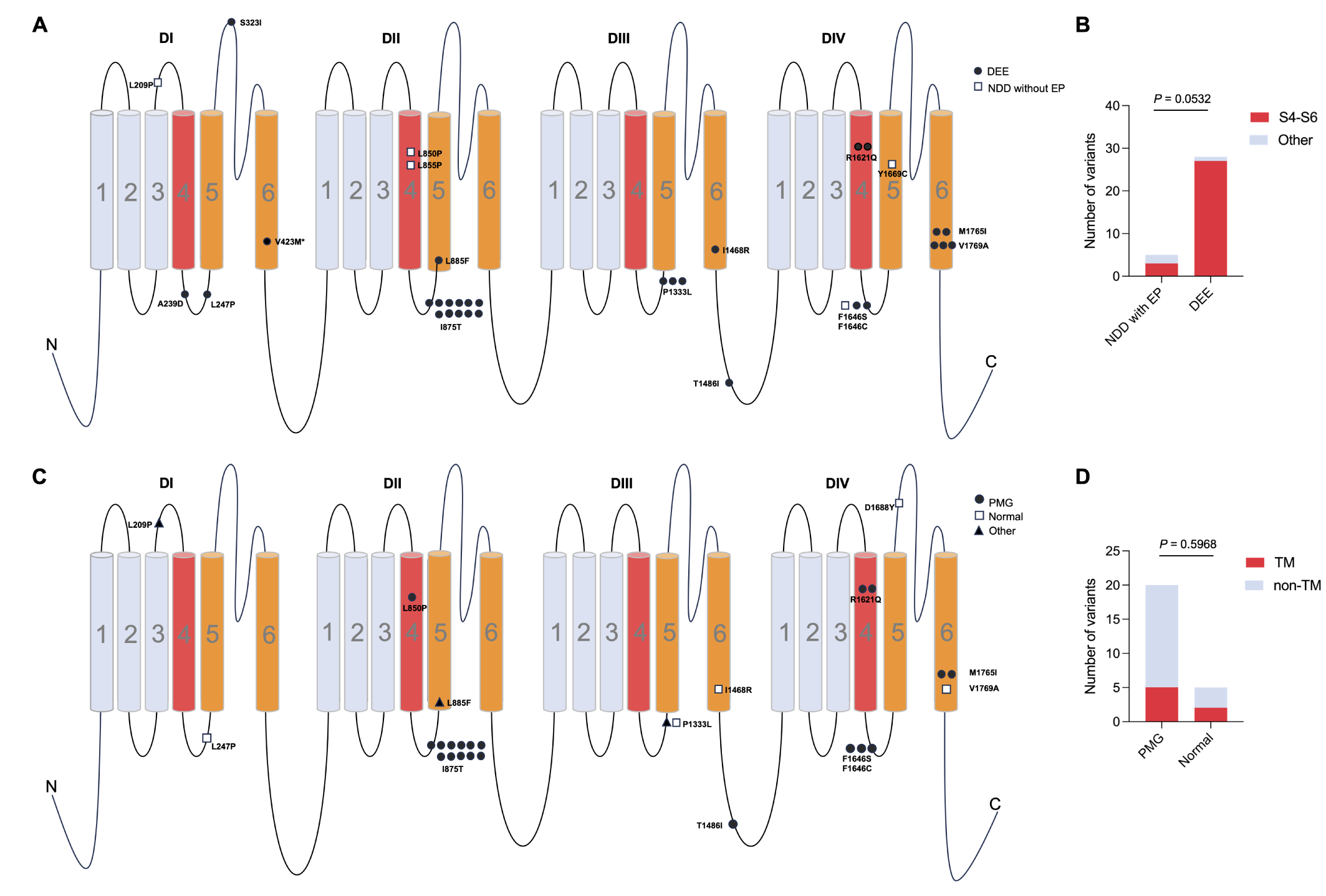

Supplement: Supplementary Figure S1 — Molecular subregional effects analysis of SCN3A variants. (A) Schematic representation of the Nav1.3 proteins structure highlighting the positions of variants associated with developmental and epileptic encephalopathy (DEE) and neurodevelopmental disorders (NDD) without epilepsy phenotype (EP). (B) Bar chart comparing the number of variants in S4-S6 segments vs. other segments for patients with NDD with EP and DEE. No significant was shown. (C) The location of variants associated with polymicrogyria (PMG), normal, and other phenotypes. (D) Bar chart comparing the number of variants in transmembrane (TM) segments vs. non-transmembrane (non-TM) segments for patients with PMG and normal individuals. No significant was shown. [file Image_1.tif]
