# Supplementary material for: Toward precision medicine in SCN3A variants-associated encephalopathies and epilepsy: optimizing genetic diagnosis and molecular subregional effects
Source: Front Neurol. 2026 Feb 5;17:1772239. doi: 10.3389/fneur.2026.1772239 (PMC12916406; doi:10.3389/fneur.2026.1772239)
Supplement: Supplementary Table S1 — Detailed description of algorithms. [file Table_1.docx]

**Table S1. Detailed description of algorithms**

| **Algorithms** | **Description** |
| --- | --- |
| AlphaMissense | Developed by Google DeepMind, this tool leverages protein structure and evolutionary information via a modified AlphaFold architecture to predict whether a missense variant is likely pathogenic or benign. |
| BayesDel_addAF | A Bayesian deleteriousness score that integrates allele frequency (AF) data with functional annotations to prioritize pathogenic variants. |
| BayesDel_noAF | Similar to BayesDel_addAF, but excludes allele frequency information, making it suitable for evaluating rare or private variants without population frequency bias. |
| ClinPred | Integrates multiple genomic features and uses a machine learning model trained on known pathogenic and benign variants from ClinVar to predict clinical relevance. |
| ESM1b | Uses embeddings from the Evolutionary Scale Modeling (ESM-1b) language model of protein sequences to assess variant effects based on evolutionary conservation and structural context. |
| fathmm-XF_coding | An extension of FATHMM that uses extreme gradient boosting to predict the functional consequences of coding and non-coding variants, including missense changes. |
| LIST-S2 | Leverages local sequence identity and structural similarity to infer the deleteriousness of missense variants, particularly effective for genes with paralogs. |
| M-CAP | Stands for Mendelian Clinically Applicable Pathogenicity; designed to prioritize rare variants with high sensitivity for Mendelian disease mutations. |
| MetaLR | A meta-predictor that combines multiple annotation scores using logistic regression to classify variants as damaging or tolerated. |
| MetaRNN | A deep learning–based meta-predictor using recurrent neural networks to integrate diverse genomic features for pathogenicity prediction. |
| MetaSVM | Another meta-predictor that uses a support vector machine to aggregate scores from multiple tools into a unified pathogenicity assessment. |
| MutationAssessor | Predicts functional impact based on evolutionary conservation of amino acid residues across homologous protein families. |
| MutationTaster | Evaluates disease-causing potential by integrating conservation, splice-site effects, and protein features using a Naive Bayes classifier. |
| Polyphen2_HDIV | PolyPhen-2 model trained on HumDiv dataset (human disease vs. common polymorphisms); optimized for distinguishing severe Mendelian disease mutations. |
| Polyphen2_HVAR | PolyPhen-2 model trained on HumVar dataset (all human disease variants vs. neutral variants); better suited for complex disease contexts. |
| PROVEAN | Predicts impact of amino acid substitutions on biological function using delta alignment scores from sequence homologs. |
| PrimateAI | A deep neural network trained on primate genome variation to distinguish benign human variants from pathogenic ones, leveraging evolutionary constraint. The input to the network is the amino acid sequence flanking the variant of interest and the orthologous sequence alignments in other species, without any additional human-engineered features, and the output is the pathogenicity score from 0 (less pathogenic) to 1 (more pathogenic). To incorporate information about protein structure, PrimateAI learns to predict secondary structure and solvent accessibility from amino acid sequence and includes these as sub-networks in the full model. The total size of the network, with protein structure included, is 36 layers of convolutions, consisting of roughly 400,000 trainable parameters. |
| SIFT | Sorting Intolerant From Tolerant, predicts whether an amino acid substitution affects protein function based on sequence homology and physical properties. |
| SIFT4G | SIFT for Genomes: an updated, faster version of SIFT optimized for large-scale genomic datasets using precomputed alignments. |
