# Supplementary material for: Toward precision medicine in SCN3A variants-associated encephalopathies and epilepsy: optimizing genetic diagnosis and molecular subregional effects
Source: Front Neurol. 2026 Feb 5;17:1772239. doi: 10.3389/fneur.2026.1772239 (PMC12916406; doi:10.3389/fneur.2026.1772239)
Supplement: Supplementary Table S2 — Performance valuation of nineteen algorithms at the recommended threshold. [file Table_2.docx]

**Table S2. Performance valuation of nineteen algorithms at the recommended threshold**

| **Algorithms** | **TP** | **TN** | **FP** | **FN** | **Accuracy** | **Balanced Accuracy** | **Sensitivity** | **Specificity** | **PPV** | **NPV** | **MCC** | **F1_score** |
| --- | --- | --- | --- | --- | --- | --- | --- | --- | --- | --- | --- | --- |
| AlphaMissense | 18 | 41 | 4 | 2 | 0.9077 | 0.9056 | 0.9 | 0.9111 | 0.8182 | 0.9535 | 0.7911 | 0.8571 |
| PrimateAI | 18 | 40 | 5 | 2 | 0.8923 | 0.8944 | 0.9 | 0.8889 | 0.7826 | 0.9524 | 0.7615 | 0.8372 |
| SIFT4G | 17 | 41 | 4 | 3 | 0.8923 | 0.8806 | 0.85 | 0.9111 | 0.8095 | 0.9318 | 0.7512 | 0.8293 |
| BayesDel_addAF | 20 | 36 | 9 | 0 | 0.8615 | 0.9 | 1 | 0.8 | 0.6897 | 1 | 0.7428 | 0.8163 |
| ClinPred | 20 | 35 | 10 | 0 | 0.8462 | 0.8889 | 1 | 0.7778 | 0.6667 | 1 | 0.7201 | 0.8 |
| MetaRNN | 20 | 30 | 15 | 0 | 0.7692 | 0.8333 | 1 | 0.6667 | 0.5714 | 1 | 0.6172 | 0.7273 |
| ESM1b | 19 | 31 | 14 | 1 | 0.7692 | 0.8194 | 0.95 | 0.6889 | 0.5758 | 0.9688 | 0.5898 | 0.717 |
| BayesDel_noAF | 20 | 28 | 17 | 0 | 0.7385 | 0.8111 | 1 | 0.6222 | 0.5405 | 1 | 0.5799 | 0.7018 |
| Polyphen2_HVAR | 15 | 37 | 8 | 5 | 0.8 | 0.7861 | 0.75 | 0.8222 | 0.6522 | 0.881 | 0.5523 | 0.6977 |
| Polyphen2_HDIV | 16 | 34 | 11 | 4 | 0.7692 | 0.7778 | 0.8 | 0.7556 | 0.5926 | 0.8947 | 0.5203 | 0.6809 |
| SIFT | 20 | 23 | 22 | 0 | 0.6615 | 0.7556 | 1 | 0.5111 | 0.4762 | 1 | 0.4933 | 0.6452 |
| MutationAssessor | 20 | 22 | 23 | 0 | 0.6462 | 0.7444 | 1 | 0.4889 | 0.4651 | 1 | 0.4769 | 0.6349 |
| PROVEAN | 20 | 21 | 24 | 0 | 0.6308 | 0.7333 | 1 | 0.4667 | 0.4545 | 1 | 0.4606 | 0.625 |
| MutationTaster | 20 | 20 | 25 | 0 | 0.6154 | 0.7222 | 1 | 0.4444 | 0.4444 | 1 | 0.4444 | 0.6154 |
| MetaSVM | 20 | 17 | 28 | 0 | 0.5692 | 0.6889 | 1 | 0.3778 | 0.4167 | 1 | 0.3967 | 0.5882 |
| LIST-S2 | 18 | 21 | 24 | 2 | 0.6 | 0.6833 | 0.9 | 0.4667 | 0.4286 | 0.913 | 0.3539 | 0.5806 |
| MetaLR | 20 | 12 | 33 | 0 | 0.4923 | 0.6333 | 1 | 0.2667 | 0.3774 | 1 | 0.3172 | 0.5479 |
| fathmm-XF_coding | 19 | 15 | 30 | 1 | 0.5231 | 0.6417 | 0.95 | 0.3333 | 0.3878 | 0.9375 | 0.3036 | 0.5507 |
| M-CAP | 20 | 8 | 35 | 0 | 0.4444 | 0.593 | 1 | 0.186 | 0.3636 | 1 | 0.2601 | 0.5333 |

Abbreviations: FN, false negative; FP, false positive; MCC, Matthews correlation coefficient; NPV, negative predictive value; PPV, positive predictive value; TN, true negative; TP, true positive.
