# Supplementary material for: Toward precision medicine in SCN3A variants-associated encephalopathies and epilepsy: optimizing genetic diagnosis and molecular subregional effects
Source: Front Neurol. 2026 Feb 5;17:1772239. doi: 10.3389/fneur.2026.1772239 (PMC12916406; doi:10.3389/fneur.2026.1772239)
Supplement: Supplementary Table S3 — Performance valuation of nineteen algorithms at the best threshold. [file Table_3.docx]

**TableS3.Performancevaluationofnineteenalgorithmsatthebest****threshold**

| **Algorithms** | **AUC** | **SE** | **95%CI** | **Best threshold*** | **Accuracy** | **Sensitivity** | **Specificity** | **PPV** | **NPV** | **MCC** | **F** |
| --- | --- | --- | --- | --- | --- | --- | --- | --- | --- | --- | --- |
| MetaRNN | 0.982 | 0.011 | 0.958-1 | 0.830 | 0.954 | 0.95 | 0.956 | 0.905 | 0.977 | 0.894 | 0.927 |
| BayesDel_addAF | 0.981 | 0.011 | 0.956-1 | 0.401 | 0.954 | 0.9 | 0.978 | 0.947 | 0.957 | 0.891 | 0.923 |
| ClinPred | 0.979 | 0.013 | 0.95-1 | 0.892 | 0.938 | 1 | 0.911 | 0.833 | 1.000 | 0.871 | 0.909 |
| AlphaMissense | 0.968 | 0.017 | 0.932-1 | 0.699 | 0.923 | 0.9 | 0.933 | 0.857 | 0.955 | 0.822 | 0.878 |
| MetaLR | 0.963 | 0.020 | 0.923-1 | 0.948 | 0.938 | 0.9 | 0.956 | 0.900 | 0.956 | 0.856 | 0.900 |
| BayesDel_noAF | 0.961 | 0.021 | 0.917-1 | 0.354 | 0.938 | 0.9 | 0.956 | 0.900 | 0.956 | 0.856 | 0.900 |
| M-CAP | 0.959 | 0.025 | 0.902-1 | 0.265 | 0.938 | 0.95 | 0.933 | 0.864 | 0.977 | 0.862 | 0.905 |
| ESM1b | 0.954 | 0.024 | 0.907-1 | -10.080 | 0.923 | 0.95 | 0.911 | 0.826 | 0.976 | 0.831 | 0.884 |
| MetaSVM | 0.952 | 0.025 | 0.904-1 | 0.975 | 0.908 | 0.95 | 0.889 | 0.792 | 0.976 | 0.802 | 0.864 |
| MutationTaster | 0.949 | 0.028 | 0.89-1 | 0.775 | 0.954 | 0.9 | 0.978 | 0.947 | 0.957 | 0.891 | 0.923 |
| SIFT | 0.948 | 0.025 | 0.898-0.998 | 0.001 | 0.923 | 0.85 | 0.956 | 0.895 | 0.935 | 0.817 | 0.872 |
| SIFT4G | 0.943 | 0.027 | 0.89-0.997 | 0.105 | 0.862 | 1 | 0.800 | 0.690 | 1.000 | 0.743 | 0.816 |
| PrimateAI | 0.912 | 0.048 | 0.812-1 | 0.856 | 0.938 | 0.9 | 0.956 | 0.900 | 0.956 | 0.856 | 0.900 |
| MutationAssessor | 0.909 | 0.041 | 0.829-0.99 | 3.138 | 0.892 | 0.8 | 0.933 | 0.842 | 0.913 | 0.744 | 0.821 |
| Polyphen2_HVAR | 0.883 | 0.042 | 0.801-0.965 | 0.443 | 0.800 | 0.95 | 0.733 | 0.613 | 0.971 | 0.631 | 0.745 |
| PROVEAN | 0.882 | 0.043 | 0.798-0.965 | -3.450 | 0.815 | 0.9 | 0.778 | 0.643 | 0.946 | 0.632 | 0.750 |
| fathmm-XF_coding | 0.863 | 0.053 | 0.76-0.967 | 0.926 | 0.862 | 0.8 | 0.889 | 0.762 | 0.909 | 0.680 | 0.780 |
| Polyphen2_HDIV | 0.859 | 0.047 | 0.767-0.951 | 0.899 | 0.785 | 0.9 | 0.733 | 0.600 | 0.943 | 0.586 | 0.720 |
| LIST-S2 | 0.806 | 0.069 | 0.67-0.941 | 0.941 | 0.831 | 0.75 | 0.867 | 0.714 | 0.886 | 0.609 | 0.732 |

Abbreviations and footnotes: *, potential best thresholds without independent external validation; Best threshold, optimal threshold corresponding to the maximum Youden index; CI, confidence interval; FN, false negative; FP, false positive; MCC, Matthews correlation coefficient; NPV, negative predictive value; PPV, positive predictive value; SE, standard error; TN, true negative; TP, true positive.
